# Location Optimization of COVID-19 Vaccination Sites: Case in Hillsborough County, Florida

**DOI:** 10.3390/ijerph191912443

**Published:** 2022-09-29

**Authors:** Yuzhou Chen, Ran Tao, Joni Downs

**Affiliations:** School of Geosciences, University of South Florida, Tampa, FL 33620, USA

**Keywords:** COVID-19, location optimization, inequality

## Abstract

The equitable allocation of COVID-19 vaccines is a critical challenge worldwide, given that the pandemic has been disproportionally affecting economically disadvantaged racial and ethnic groups. In the United States, the ongoing implementation efforts at different administrative levels and districts, to some extent, are standing in conflict with commitments to mitigate inequities. In this study, we developed a spatial optimization model to choose the best locations for vaccination sites. The model is a modified two-step maximal covering location problem (MCLP). It aims at maximizing the number of residents who can conveniently access the sites and mitigating inequity issues by prioritizing disadvantaged population groups who live in geographic areas identified through the CDC’s Social Vulnerability Index (SVI). We conducted our study using the case of Hillsborough County, Florida. We found that by reserving up to 30% of total vaccines for highly vulnerable communities, our model can optimize location choices for vaccination sites to provide effective coverage for residents at large while prioritizing disadvantaged groups of people. A series of sensitivity analyses have been performed to evaluate the impact of parameters such as site capacity and distance threshold. The model has the potential to guide the future allocation of critical medical resources in the U.S. and other countries.

## 1. Introduction

The COVID-19 pandemic has been disproportionally affecting different groups of people in the world. People of low socio-economic status are particularly vulnerable to COVID-19 because of factors including their usually overcrowded living conditions, lack of opportunities to work in a safe environment, lack of access to proper healthcare services, etc. [1]. Therefore, to guarantee a sufficient and equitable vaccine allocation is a top priority for many countries. In the U.S., the *National Strategy for the COVID-19 Response and Pandemic Preparedness* [2] released by the Biden administration in January 2021 specifically addressed this matter: “the United States will work to ensure that the vaccine is distributed quickly, effectively, and equitable, with a focus on making sure that high-risk and hard-to-reach communities are not left behind”. In terms of implementation, a common strategy recommended by the National Academies of Sciences, Engineering, and Medicine [3] takes two steps: providing available doses to jurisdictions according to their population and then distributing vaccines among specific populations within jurisdictions [4]. The specific population dealt with in the second step are the worst-off income and racial groups that are more vulnerable to COVID-19. To identify where they live, NASEM recommended the use of geographic areas identified through the CDC’s Social Vulnerability Index (SVI) or another more specific index, and, for example, setting aside 10% of federally available vaccines for high-SVI communities. However, in early 2021 only 18 jurisdictions in the U.S. indicated using a disadvantage index for planning the location of vaccination sites or communication and outreach efforts. For example, Tennessee, New Hampshire, North Carolina, and California reserve a certain amount of their vaccines for high-SVI areas, ranging from 5% to 40%. However, jurisdictions like Nevada and Mississippi did not have such a plan, despite over a quarter of their populations living in high-SVI communities [4]. To make matters worse, actions taken by governments at different administrative levels are not thoughtfully coordinated. It is questionable how well the equitable allocation of vaccines has been carried out in such a short period of time. For instance, while some regions are served simultaneously by vaccinations sites operated by the federal, state, and county governments, some other regions are deserts of vaccination services. It is important to evaluate the sufficiency and equity provided by the existing vaccination sites and to propose improvement strategies by choosing better locations. 

Cabanilla et al. [5] proposed an approach to determine the optimal location of vaccination sites based on minimizing the distance between sites and administrative units. They test the number of vaccination sites from one to four, and the results show that four vaccination sites have the best performance. Bertsimas et al. [6] developed a DELPHI-based coordinate descent algorithm to optimize the location of vaccination sites and subsequent vaccine allocation. The results showed that the method increased the effectiveness of vaccination campaigns by about 20%. However, the previous models did not consider that people should be covered more than once. In fact, people should be covered multiple times with different vaccination sites in terms of their needing a second or boost dose, or everyone should be covered by various vaccination sites.

In this paper, we developed a spatial optimization model to choose the best locations for COVID-19 vaccination sites, demonstrated by a case study in Hillsborough County, Florida, where equitable allocation of critical medical resources was lacking [7]. The impacts and sensitivity of important parameters were discussed.

## 2. Method

### 2.1. Study Area and Data Collection

Our study area is Hillsborough County, Florida, USA. With around 1.5 million residents, the county is the fourth-largest county in Florida by population. The county seat is the city of Tampa. The basic spatial units of our analysis are the 316 census tracts in the county. In May 2021, there were 289 COVID-19 vaccination sites operated in Hillsborough County. These sites were managed by four categories of parties or agencies: federal, state, county, and others. Specifically, five sites were operated by federal agencies like the Federal Emergency Management Agency (FEMA), including one large site and four medium sites in terms of capacity. The large site was set up at the Tampa Greyhound Track and it could conduct about 2000 vaccinations per day. The medium sites had a daily capacity of 500, and their locations rotated weekly. The state government of Florida operated one mass vaccination site at the Raymond James Stadium with a daily capacity of 2500. Hillsborough County operated three open points of dispensing (PODs) sites and each of them could conduct 1000 vaccinations per day. Finally, there were 282 sites operated by other parties such as hospitals, clinics, pharmacies, and grocery stores like Walmart and Publix. However, the capacity of these sites varies considerably, and the exact numbers are difficult to track. In our model, we assign a daily capacity of 50 for all sites run by other parties, estimated by the online appointment scheduling system of CVS and Walgreen (the two largest chain pharmacy stores in the U.S. that offer COVID-19 testing and vaccination services).

**Figure 1:**
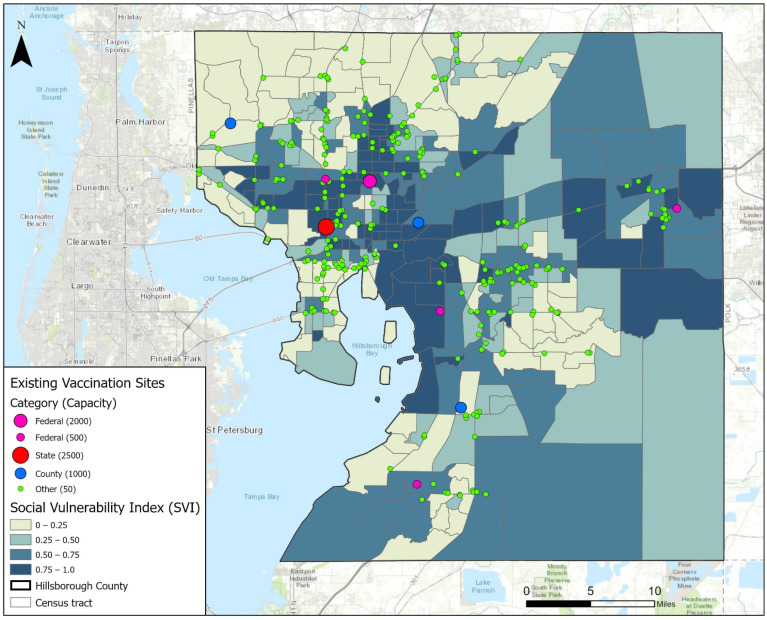
Existing vaccination sites in Hillsborough County, Florida.

Figure 1 displays the locations of the existing vaccination sites in May 2021, which was at the peak rate during the Biden administration’s campaign to vaccinate 70% of American adults by 4 July 2021. The spatial distribution is rather uneven as most sites are clustered in the city of Tampa that is north of the shore of Old Tampa Bay and in Brandon that is east of the shore of Hillsborough Bay. Referring to the base map, which shows CDC’s Social Vulnerability Index (SVI) at the census tract level, the 289 vaccination sites failed to sufficiently cover many highly vulnerable areas. For instance, the dark regions near the geographic center and in the eastern part of the county had very few sites operated there. It raises questions about whether the existing vaccine allocation strategy is equitable. The complexity of allocating and distributing vaccines is extremely high. There are inevitable issues caused by the lack of communication and resource sharing among different operators and the different protocols in terms of priority, qualification, scheduling, etc. 

**Figure 2:**
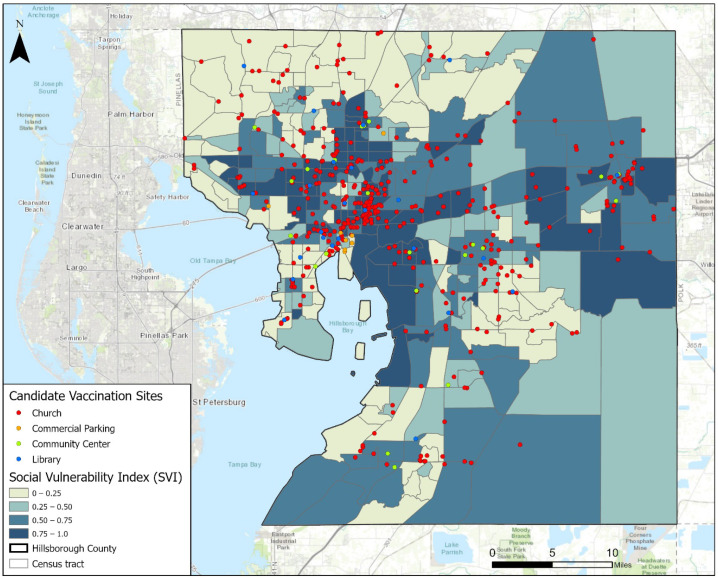
Candidate vaccination sites in Hillsborough County, Florida.

To improve the allocation of vaccines, we searched for better locations to set up new vaccination sites that can replace some of the existing ones. By consulting the domain experts from Hillsborough County Emergency Management, we came up with the criterion for a candidate vaccination site: a relatively large open space for setting up a POD site (a specialized vehicle and several tents) that can be borrowed or rented for several days of a week. Based on this criterion, we have found 484 candidate locations: 430 churches’ park lots, 22 community centers, 24 public libraries, and 8 commercial parking lots. Figure 2 shows the locations of these candidate vaccination sites. In contrast with the existing sites displayed in Figure 1, the candidate sites are spread more evenly in space. A considerable number of candidate sites are located in the highly vulnerable regions, which can potentially help mitigate the inequity issue in accessing vaccines. 

### 2.2. Two-Step MCLP Model

Inspired by the general strategy recommended by NASEM, i.e., to reserve a certain number of vaccines for the vulnerable population, we designed a two-step spatial optimization model by extending the classic maximal covering location problem (MCLP) [8]. In short, an MCLP model aims to maximize the coverage of space by choosing a fixed number of service locations from the pool. Using similar logic, our model also has a main objective, which is to maximize the population covered by the service area of vaccination sites through choosing the optimal locations from the pool of existing and candidate locations. We used the census survey data to divide the population into two groups based on household car ownership because it is a deciding factor of how far people in Florida can and are willing to travel to get vaccinated. For each vaccination site, we delineate two types of service areas based on the transportation mode. Car owners can choose to drive or walk to a vaccination site, whereas people without a car can only walk to a nearby site. Although there exist other means to access a vaccination site, such as taxis and ride-hailing services, we only consider driving, walking, and public transportation in our models, given their representativeness and reliability during the pandemic. 

Objective:

(1) $$Maximize\;Z=\sum_{\forall j\in J1\cup J2}(x_{j}p_{j})$$

$$S.T.\;\;\;\;\;\;\;\sum_{i\in N_{j}}y_{i}\geq \;x_{j}\;for\;all\;j\;\in J$$

$$y_{i}=(0,1)\;\mathrm{for}\;\mathrm{all}\;i\in I$$

$$N_{j}=\{i\in I|d_{ij}\leq T\}\;$$

where *Z* is the objective index; *j* is a census tract; $J_{1}$ denotes the set of census tracts of the population with private vehicles; $J_{2}$ denotes the set of census tracts of the population without private vehicles; $y_{i}$ is the vaccination site; $N_{i}$ is a set of vaccination sites eligible to provide “cover” to census tract x; $d_{ij}$ is the distance between vaccination site *i* to the centroid of census tract *j*; *T* is the traveling threshold which is 10 min driving for people with private cars and 5 min walking and 15 min public transit for people without cars; and $S_{j}$ is the capacity of vaccination site; 

subject to: 

Constraint A: the sum of the capacity of all selected sites does not exceed *S*:

(2) $$\sum_{i=1}^{I}S_{i}y_{j}\leq S$$

where $S_{i}$ is the capacity of vaccination site *i*; *I* denotes the set of locations of both existing and candidate vaccination sites in the study area; and $y_{j}\;$ equals 1 if location *j* is selected for setting up a vaccination site, otherwise, 0; constraint A serves as a total limit of resources, namely the number of vaccines distributed per day. If setting S is the same as the current total capacity, the model would aim at redistributing the existing amount of vaccine resources at more optimal locations. Plus, it allows us to compare model results with the current deployment. Changing the value of S is meaningful when responding to varied scenarios such as a surge of demand. 

Constraint B: each census tract cannot be served more than $D_{j}/M$ times to avoid the overconcentration problem.

(3) $$\sum_{j\in J}x_{j}\leq D_{j}/M$$

where J is the set of all census tract;$\;D_{j}$ is the population density in census tract *j*. Constraint B is to avoid overconcentration of the selected locations and the double-count issue, e.g., too many sites clustered in high-SVI populous areas and each tract is to be covered too many times. The vaccination site allocation problem has the unique feature that it is inadequate for people just to be covered once. Not only does the vaccine need several doses, but also it is necessary for people to have options for different vaccination sites. Therefore, we use the population density of each census tract to set up a reasonable number of covers. The threshold $D_{j}/M$ is in proportion to the population density of census tract *j*, i.e., one vaccination site per *M* population density. 

Constraint C: the number of sites selected from candidate locations is within the range of ($\underline{L}$, $\bar{L}$):

(4) $$\underline{L}\leq \;\sum_{\forall j\in J_{2}}X_{j}\leq \bar{L}$$

where $\underline{L}$ and $\bar{L}$ are the lower bound and upper bound, respectively. Constraint C is to limit the extent of change from the current deployment, since adding more sites from the candidate locations demands a higher cost and the likely cancellation of existing sites. Assigning a small value to $\bar{L}$ would lead to a more pragmatic solution, as it limits the number of sites to be set up at new locations. 

Constraint D: the total number of selected sites is within the range of ($\underline{N}$,$\bar{N}$):

(5) $$\underline{N}\leq \sum_{\forall j\in J1\cup J2}X_{j}\leq \bar{N}$$

where $\underline{N}$ and $\bar{N}$ are the lower bound and upper bound, respectively. Constraint D is to avoid extreme solutions, e.g., many vaccination sites with small capacity, or only a few sites with large capacity.

Our two-step model is to repeatedly run the MCLP model in two consecutive steps, one for the highly vulnerable population and one for the rest of all vaccine-applicable populations. We reserve *ε*% of the total vaccines for the highly vulnerable population. Using the recommendation by NASEM, we use high-SVI (above 0.75, or the highest quantile) census tracts to represent where the vulnerable populations reside. 

In our first-step MCLP model, we use (1−ε%)∗S as the daily vaccine capacity in constraint A and select the locations from both the existing sites and candidate sites to maximize objective value. In other words, we try to cover as many people as possible, regardless of their demographic profile or socioeconomic status. In the second step, we use the reserved ε%∗S as the daily vaccine capacity in constraint A to take care of the population from the high-SVI census tracts only. The order of the two steps is logical rather than temporal, which means it does not imply that the more vulnerable population gets vaccinated after the others. However, the logical order makes a difference in the results, and we describe the details in the next section.

## 3. Results and Discussions

### 3.1. Reserved Vaccines for the Vulnerable

Table 1 lists five batches of results for comparison. Case 0 is the real situation in May 2021 with the existing 289 sites, and other cases are results of our model with different percentages of vaccines reserved for the high-SVI communities. We set *S* in constraint A the same as the real situation for all cases, namely 23,500 vaccines per day in total, to ensure the results are comparable. This means the result differences are strictly due to the choice of vaccination site locations. We set *M* in constraint B as 300, which turned out to be a balanced parameter value according to our tests. Its sensitivity is discussed in the next section. Constraints C and D are to control the degree of location changes from the existing situation. This is particularly useful when considering the complexity of reality, such as limited budgets and human resources that restrict the number of new sites to be set up, or ongoing contracts with vaccination partners that prevent them from being discontinued. Since we want to first seek a theoretically solution, we did not enforce constraints C and D except for one rule: preserve the two largest sites at the Tampa Greyhound Track and the Raymond James Stadium. The rationale is that these two sites were operated by the federal and state governments and their unique settings can hardly be replaced by any candidate locations in the county. Therefore, an extreme situation is that only the two largest existing sites are kept while all other sites are selected from the candidate locations. The opposite extreme situation is the same as Case 0: to stick with the real situation by making no change to site locations. We set the *T* to 10 min driving and 5 min walking after consulting domain experts who are in charge of vaccine distribution in Hillsborough County. In addition, we apply another *T* to 15 min public transit for people without cars so the “walking” population has a better opportunity to take the vaccine. There are two types of indicators of our model’s performance in terms of sufficiency and equity. The *Z* value in Equation (1) is used to evaluate how sufficient the vaccination sites can provide accessible service to the general population. A higher *Z* value means there are more residents covered by the service area of vaccination sites (each person can be counted *n* times if covered by *n* different sites). To assess the equity of location choices, we adopt Pearson’s correlation. The last two columns in Table 1 are the correlation between the SVI value of a census tract and the number of vaccination sites its residents can access to via driving and walking, respectively. A higher correlation between SVI and the number of walk-accessible sites (SVI~Walk) can reflect a more equitable site distribution, given that SVI is positively associated with the percentage of carless households.

As the benchmark for comparisons, Case 0 yields a *Z* value above 15.57 M (million), SVI~Drive as 0.191, and SVI~Walk as −0.03, which is N/S (non-significant). Through optimizing the locations with our model, Cases 1 to 5 have increased the *Z* value by at least 4%, indicating an obvious boost of coverage sufficiency. SVI~Drive shows a significant increase from Case 0 through all tests. In contrast, SVI~Walk has become statistically significant and positive, ranging from 0.370 to 0.381. It indicates that the results of our model are more equitable than the real situation by taking care of the carless population.

The percentage of vaccines reserved for the high-SVI communities is a key factor of model results. By reserving no vaccine for the vulnerable population, Case 1 is essentially a one-step MCLP model that maximizes the coverage of the general population. It yields the largest *Z* value among all cases, but it moderately increased SVI~Walk to 0.376. From Case 1 to Case 4, we increase the reserved vaccine for the second step from 0% to 30%. A nonlinear trend can be observed in both *Z* value and SVI~Walk: *Z* slightly decreases from 16.46 M to 16.19 M, while SVI~Drive increases from 0.227 to 0.255. SVI~Walk showed a fluctuation, which is still much better than the real situation. We argue that there is no clear winner among all tests with various reserved vaccines. If the priority is to maximize coverage, Case 1 would be the choice. On the other hand, Case 4 would be chosen if equity is also a top priority. Since Florida is a state with over a quarter of its population living in high-SVI communities [4], reserving a considerable number of vaccines for the most vulnerable communities should definitely be a top priority. Case 5 was tested to compare with Case 4. The only difference is that Case 5 uses the reserved 30% of vaccines in the first step of our model, rather than the second step like Case 4. The results show that Case 5 has the smallest *Z* value and SVI~Walk coefficient, which proves Case 4 is the most balanced result. 

**Figure 3:**
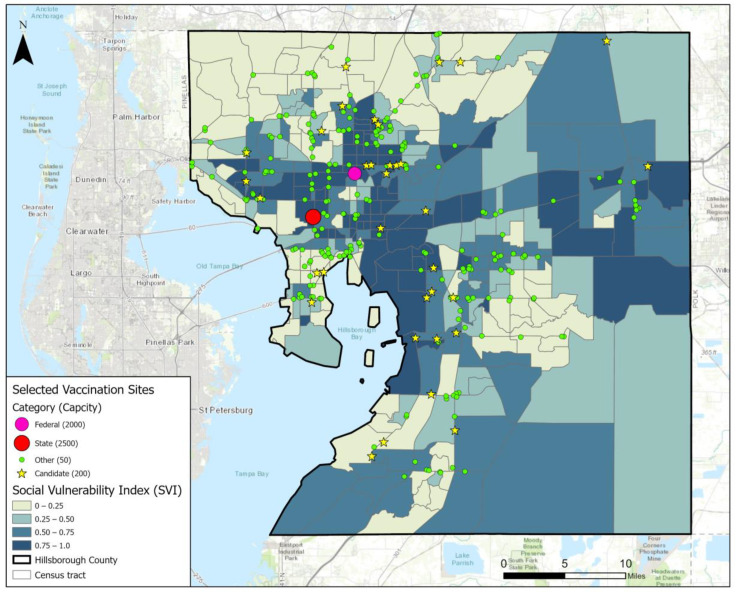
Case 4 result map with selected vaccination site locations.

Figure 3 shows the locations of selected sites by Case 4. In contrast with Case 0 in Figure 1, Case 4 introduced some new vaccination sites from the candidate locations, the majority of which were placed near the county center where population density and SVI are both high. Among the existing sites, a considerable number of small sites run by other parties were replaced by candidate sites, especially in peripheral areas such as the southern and northern parts of the county. It seems that using the candidate sites with a daily capacity of 200 is more efficient than using the small sites with a daily capacity of 50. However, the exact impact of site capacity remains to be tested. Besides, the three county-operated sites with a daily capacity of 1000 were ditched without adding new sites nearby. This might be due to undesirable locations having been chosen by the county operator. 

### 3.2. Constraint B

**Figure 4:**
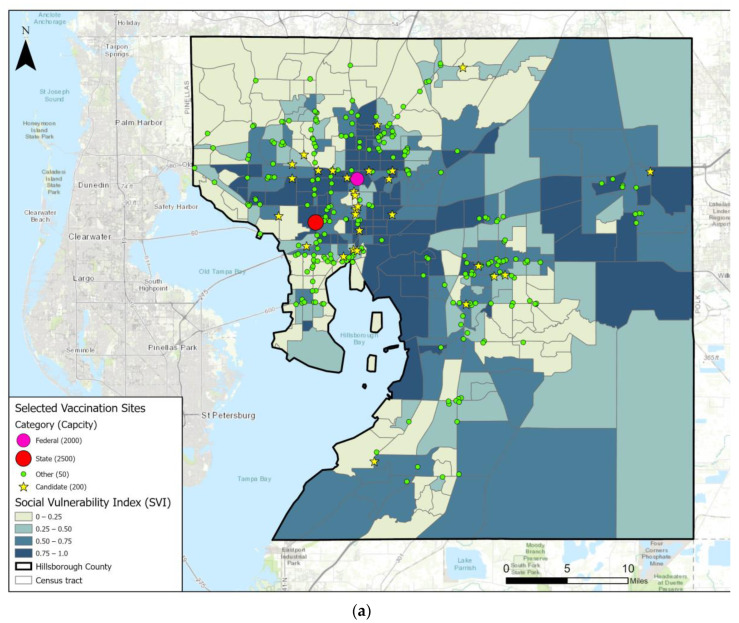

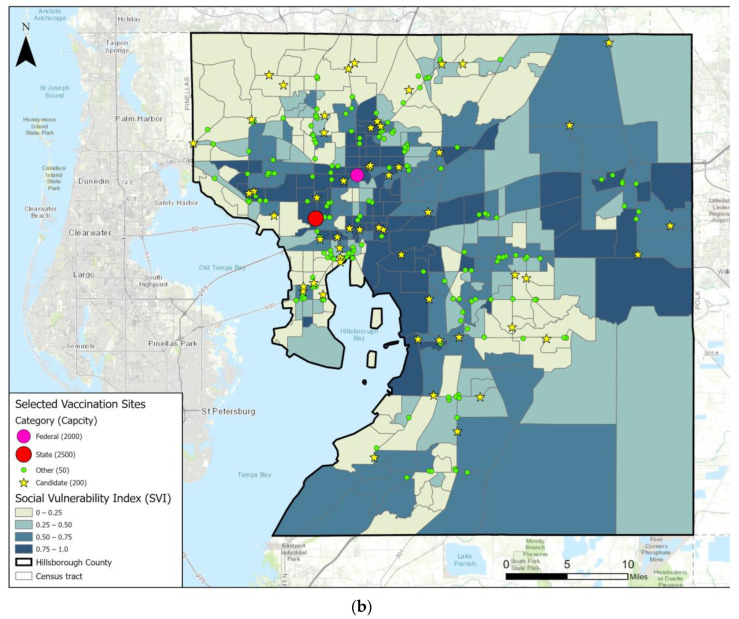
Case (**a**,**b**) result maps with selected vaccination site locations.

Constraint B is designed to avoid overconcentration of the selected locations. According to Equation (2), *M* is in proportion to the population density of a census tract. When *M* is assigned with a small value, each census tract is allowed to be covered more times. When *M* is assigned with a large value, the fewer cover times are assigned to each census tract. Table 2 lists three cases with different *M* values, while holding all other parameters the same. Case 4a and 4b set *M* as 100 and 500, respectively. Case 4 is the same as the one in Table 1 with *M* set as 300. The results in Table 2 show that Case 4 has the SVI~Walk values among the three. The different *M* will cause different Z values for the real situation case so that the Z value in Table 2 cannot be compared directly. The corresponding Z values of these three cases are 25,690,357, 15,571,906, and 10,068,676, respectively. Case 4a has the highest SVI~Drive value. However, Case 4a achieved this great performance by overly concentrating the selected locations. Figure 4a shows that the locations of selected sites by Case 4a are heavily clustered in the central areas, whereas the northern and southern parts of the county are barely served. On the other hand, Figure 4b shows that the locations selected by Case 4b are more evenly distributed in the county, but the SVI~Drive and SVI~Walk values make Case 4d suboptimal. In contrast, Case 4 with *M* set as 300 stands out as a more balanced choice. 

### 3.3. Capacities of Candidate Sites

To evaluate the impact of candidate site capacity, we generated the list of results shown in Table 3. Case 4 is still used as the benchmark or comparison, which sets the capacity of all candidate sites as 200. Cases 4c, 4d, and 4e modify capacity to 50, 500, and 1000, respectively. With the smallest capacity, Case 4c yields the highest *Z* value and SVI~Drive&Walk values, but it achieves that by replacing over half of the existing sites and building over 200 new candidate sites. By gradually increasing the capacity, the *Z* value decreases from 16.95 M in Case 4c to 15.40 M in Case 4e. In the meanwhile, SVI~Walk decreases from 0.395 in Case 4e to 0.371 in Case 4e. The impact of candidate site capacity is similar to the percentage of reserved vaccines discussed in Section 3.1. There is no absolute optimal value for this parameter, though we can argue that a reasonable choice would be between 50 to 200 to balance coverage sufficiency and equity. In the real situation, the capacity of candidate sites is more likely to be constrained by other factors such as budget, space, and human resources. For example, setting up small-capacity sites would be more demanding on personnel and logistics, while setting up big-capacity sites would require large open spaces that are challenging to find. 

### 3.4. Constraints C and D 

**Figure 5:**
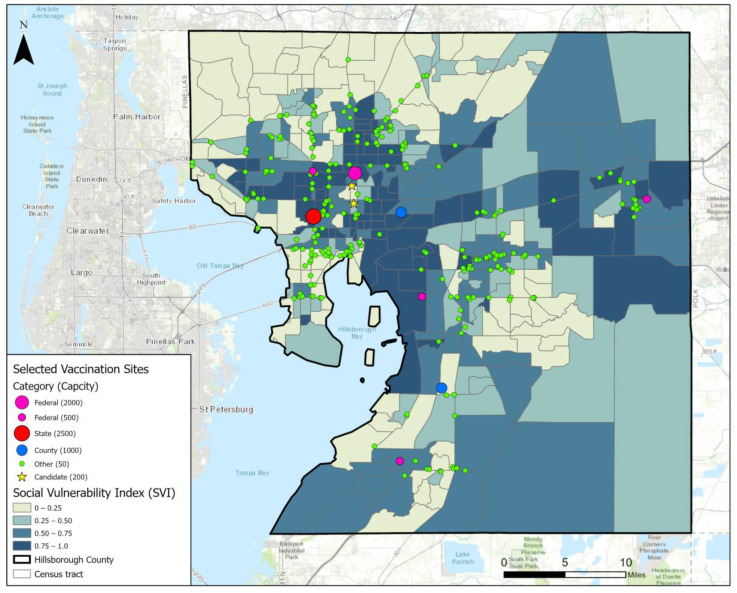
An example of practical solution by involving constraints C and D.

In all the results discussed so far, neither constraint C nor constraint D has played an effective role in the model. This is because these two constraints were designed to limit the degree of changes from the existing situation that were due to factors such as limited budget, personnel, and space that are not accounted for in our model. Nevertheless, constraints C and D are still meaningful when the goal is to achieve a more practical solution. For instance, the solution of Case 4 is to select 234 out of 289 existing sites and set up 37 news sites at the candidate locations. The degree of change is considerably big, and the difficulty of implementation is high in a short amount of time. To give an example of a practical solution, we set constraints C and D to limit the maximum number of existing locations to be removed to 10, and the maximum number of candidate locations to be added to 2. We obtained the *Z* value as 15.59 M and SVI~Walk as 0.361. Both indicators are worse than most results discussed previously without involving constraints C and D. Figure 5 displays the selected location of this practical solution. Only 10 existing locations that are operated by the non-government third parties were replaced, and two newly added sites selected from the candidate locations were placed close to each other—both sit at the city center of Tampa, which can cover the most high-SVI and populous census tracts. 

## 4. Conclusions

In this study, we developed a spatial optimization model of location selection for COVID-19 vaccination sites. We proposed a modified two-step maximal covering location problem. The model balances between maximizing the number of residents served by the vaccination sites and mitigating inequity issues by prioritizing disadvantaged groups of people. We conducted a case study in Hillsborough County, Florida. The results show that reserving a certain number of vaccines for the high-SVI communities in the second step of our model can promote a sufficient and equitable vaccine distribution. The recommended percentage of vaccine reservation is 30% in the study area. The impacts of important parameters are thoroughly discussed with sensitivity analysis. Distributing the reserved vaccines to high-SVI communities in the second step works better than in the first step. Constraint B is useful to avoid overconcentration of vaccination sites. Candidate site capacity needs to be carefully decided to avoid being too small or too big. At last, constraints C and D can help seek a practical solution by purposely limiting the number of existing sites to be removed and candidate sites to be added. 

The study has certain limitations to be addressed by future works. The current model is not time sensitive as it calculates a one-time solution to optimize vaccination site locations. However, the real situation is complex and is evolving rapidly. The optimal solution calculated at one time can quickly become less desirable when factors like vaccine production and logistics and willingness to get vaccinations change over time. Another limitation is that the model offers the optimal solution by consolidating available resources at different administrative levels and private sectors, which can be challenging in terms of implementation. Nevertheless, the model sets an example of optimizing locations of COVID-19 vaccination sites, which can be a useful guide to future allocation of critical medical resources. 

## Figures and Tables

**Table 1:** Model results with different amounts of reserved vaccines.

Case No.	Description	*S* in Constraint A	*M* in Constraint B	Selected Existing Sites	Selected Candidate Sites	*Z* in Objective	SVI~Drive	SVI~Walk
0	The real situation	23,500	300	289	0	15,571,906	0.191	−0.03 (N/S)
1	0% Reserved	23,500	300	234	37	16,463,086	0.227	0.376
2	10% Reserved	23,500	300	239	37	16,432,874	0.229	0.381
3	20% Reserved	23,500	300	242	35	16,346,930	0.238	0.376
4	30% Reserved	23,500	300	234	37	16,203,260	0.255	0.381
5	30% Reserved for step1	23,500	300	238	36	16,199,157	0.218	0.370

**Table 2:** Model results with different *M* values in constraint B.

Case No.	Description	*S* in Constraint A	*M* in Constraint B	Selected Existing Sites	Selected Candidate Sites	*Z* in Objective	SVI~Drive	SVI~Walk
4a	30% Reserved	23,500	100	254	32	29,741,147	0.264	0.373
4	30% Reserved	23,500	300	234	37	16,203,260	0.255	0.381
4b	30% Reserved	23,500	500	162	55	10,344,833	0.238	0.377

**Table 3:** Model results with different candidate site capacities.

Case No.	Description	*S* in Constraint A	*M* in Constraint B	Candidate Site Capacity	Selected Existing Sites	Selected Candidate Sites	*Z* in Objective	SVI~Drive	SVI~Walk
4c	30% Reserved	23,500	300	50	112	212	16,956,177	0.305	0.395
4	30% Reserved	23,500	300	200	234	37	16,203,260	0.255	0.381
4d	30% Reserved	23,500	300	500	242	14	15,568,174	0.241	0.376
4e	30% Reserved	23,500	300	1000	232	7	15,407,636	0.232	0.371
